# The Potential of Lisosan G as a Possible Treatment for Glaucoma

**DOI:** 10.3389/fphar.2021.719951

**Published:** 2021-07-28

**Authors:** Rosario Amato, Maria Grazia Rossino, Maurizio Cammalleri, Anna Maria Timperio, Giuseppina Fanelli, Massimo Dal Monte, Laura Pucci, Giovanni Casini

**Affiliations:** ^1^ Department of Biology, University of Pisa, Pisa, Italy; ^2^ Interdepartmental Research Center Nutrafood “Nutraceuticals and Food for Health”, University of Pisa, Pisa, Italy; ^3^ Department of Ecological and Biological Sciences, University of Tuscia, Viterbo, Italy; ^4^ Department of Agriculture and Forest Sciences, University of Tuscia, Viterbo, Italy; ^5^ National Research Council, Institute of Agricultural Biology and Biotechnology (IBBA), Pisa, Italy

**Keywords:** inflammation, neuroprotection, nutraceuticals, oxidative stress, pattern electroretinogram

## Abstract

Lisosan G (LG), a fermented powder obtained from whole grains, is a nutritional supplement containing a variety of metabolites with documented antioxidant properties. We have recently demonstrated that orally administered LG protects diabetic rodent retinas from oxidative stress, inflammation, apoptosis, blood-retinal barrier disruption, and functional damage. Here, we investigated whether LG may exert protective effects in a model of glaucoma and measured the amounts of selected LG components that reach the retina after oral LG administration. Six-month-old DBA/2J mice were given an aqueous LG solution in place of drinking water for 2 mo. During the 2 mo of treatment with LG, the intraocular pressure (IOP) was monitored and the retinal ganglion cell (RGC) functional activity was recorded with pattern-electroretinography (PERG). At the end of the 2-mo period, the expression of oxidative stress and inflammatory markers was measured with qPCR, and RGC survival or macroglial activation were assessed with immunofluorescence. Alternatively, LG was administered by gavage and the concentrations of four of the main LG components (nicotinamide, gallic acid, 4-hydroxybenzoic acid, and quercetin) were measured in the retinas in the following 24 h using mass spectrometry. LG treatment in DBA/2J mice did not influence IOP, but it affected RGC function since PERG amplitude was increased and PERG latency was decreased with respect to untreated DBA/2J mice. This improvement of RGC function was concomitant with a significant decrease of both oxidative stress and inflammation marker expression, of RGC loss, and of macroglial activation. All four LG metabolites were found in the retina, although with different proportions with respect to the amount in the dose of administered LG, and with different temporal profiles in the 24 h following administration. These findings are consistent with neuroenhancing and neuroprotective effects of LG in glaucoma that are likely to derive from its powerful antioxidant properties. The co-occurrence of different metabolites in LG may provide an added value to their beneficial effects and indicate LG as a basis for the potential treatment of a variety of retinal pathologies.

## Introduction

Oxidative stress and inflammation can be considered as pathogenetic hallmarks of a variety of retinal diseases, including age related macular degeneration, diabetic retinopathy, retinitis pigmentosa, and glaucoma. Regarding glaucoma, although elevated intraocular pressure (IOP) is commonly considered the main risk factor for this disorder (Gottanka et al., 2005). IOP lowering is not always effective in preventing disease progression, and the form of glaucoma known as normotensive glaucoma develops with the IOP within the normal range, indicating a high degree of complexity in the pathophysiology of this retinal disease (Weinreb et al., 2014). Indeed, a variety of investigations have highlighted the primary role played by oxidative stress and inflammation in the development of glaucoma (Baudouin et al., 2020; Domènech and Marfany, 2020; Garcia-Medina et al., 2020; Mélik Parsadaniantz et al., 2020; Wang et al., 2020), indicating that potential treatments of this sight-threatening disease, characterized by retinal ganglion cell (RGC) degeneration and visual field loss, could be based on the use of antioxidant and/or anti-inflammatory compounds.

Among the various treatment options that may be considered for glaucoma, functional foods and nutraceuticals are particularly attractive. In view of their availability, their ease of use as natural dietary supplements, and the lack of induced collateral side effects (Kalra, 2003; Chauhan et al., 2013), these substances may constitute a strong basis to develop novel drugs for the treatment of this disease (Adornetto et al., 2020) as well as of other retinal pathologies (Rossino and Casini, 2019). The potential of nutraceuticals lies in their powerful antioxidant effects. Indeed, exogenous antioxidants of natural origin may be used to preserve redox homeostasis acting directly as scavengers of free radicals, indirectly by interrupting free radical chain reactions, or both. They may also decrease oxidative stress by inducing the expression of endogenous antioxidant enzymes (Nimse and Pal, 2015; Ahmadinejad et al., 2017). An antioxidant action of nutraceuticals may also reduce inflammation. Indeed, oxidative stress has been recognized as playing a pivotal role in the development of inflammation (Gill et al., 2010; Reuter et al., 2010), which, in glaucoma, would induce macroglial cell activation, characterized by increased glial fibrillary acidic protein (GFAP) expression in Müller cells (Mélik Parsadaniantz et al., 2020), and a marked release of cytokines/chemokines that would damage retinal neurons (Chen et al., 2010).

We have shown recently that Lisosan G (LG), a fermented powder obtained from organic whole grains (*Triticum aestivum*) and registered with the Italian Ministry of Health as a nutritional supplement, protects the retina from oxidative stress and significantly reduces inflammation and the retinal damage associated to diabetic retinopathy (Amato et al., 2018). Its powerful antioxidant properties are likely to be due to a rich variety of compounds, as LG has been shown to contain bioactive substances such as both flavonoid and non-flavonoid polyphenols, alpha-lipoic acid, polyunsaturated fatty acids, and vitamins, among others (La Marca et al., 2013; Lucchesi et al., 2014).

In the present paper, we tested whether LG has the potential to be considered as a valid option for the treatment of glaucoma using the DBA/2J (D2) mouse model, in which the spontaneous increase in IOP starting at 6 mo of age correlates with a glaucomatous age-related RGC loss (Schnichels et al., 2021). To this aim, in D2 mice, the effects of LG were tested on IOP levels, on RGC function and survival, on the expression of oxidative stress and of inflammatory markers, and on macroglial activation. In addition, selected components of LG were assessed in the retina using mass spectrometry.

## Materials and Methods

### Animals

All the procedures were performed in compliance with the ARVO Statement for the Use of Animals in Ophthalmic and Vision Research, the EU Directive (2010/63/EU), and the Italian guidelines for animal care (DL 26/14; Permission number: 349/2018-PR). A total of 34 D2 mice of both sexes (Charles River Laboratories, Calco, Italy) were used in these studies. They were kept in a regulated environment (23 ± 1°C, 50 ± 5% humidity) with a 12 h light/dark cycle (lights on at 8:00 a.m.) with food and water (or a LG solution, *see* below) ad libitum. Non-glaucomatous control mice were not considered since the commonly used C57BL/6J or DBA/2J-Gpnmb+/SjJ show some notable differences in inner retinal neural processing that could have a counterpart in the RGC susceptibility to insults or diseases (Porciatti et al., 2010). Therefore, we focused on differences between LG-treated and untreated DBA/2J mice without introducing a possibly biased, non-glaucomatous control.

### Preparation of LG and LG Administration

LG was supplied by Agrisan Company (Larciano, Pistoia, Italy). It is a powder obtained by fermenting and drying whole wheat flour from *Triticum aestivum* grains. The starter cultures typically consist of a mix of lactobacillus and natural yeast strains in a ratio of about 100:1 (Natural Sourdough). Once the product was fermented, it was dried using a vacuum pump at 20–25°C temperature and 2 bar pressure until reaching 12% humidity (48–60 h for 100 kg material).

LG was administered in the form of an aqueous solution of the hydrophilic components of LG. Twelve D2 mice were randomly distributed among control or LG-treated groups (*n* = 6 for each experimental group). The D2 mice used as controls had ad libitum access to food and water, while, for the D2 mice treated with LG (D2+LG), water was replaced with the LG solution. The treatment began at 6 mo of age (baseline), and it was continued for 2 mo (8 mo of age, endpoint). At the end of this period, the mice were sacrificed, and the retinas were dissected and used for molecular and immunohistochemical analyses.

For the analysis of different LG components reaching the retina after LG ingestion, a total of eighteen 6-mo-old D2 mice received an oral administration of LG solution by gavage. The retinas of three mice were dissected and quickly frozen in liquid nitrogen after 0 or 30 min and after 1, 2, 6, or 24 h. The retinas were stored at −80°C until used for mass spectrometry.

The aqueous LG solution had a concentration of 8.3 mg/ml. Preliminary observations showed that the volume of LG solution consumed by these mice was similar to that of drinking water consumed by untreated D2 mice (about 3 ml/mouse/day). Assuming an average mouse body weight of 20 g, this dose corresponded to 1 g LG/Kg/day. This dose was equivalent to that used in previous studies in rats (Longo et al., 2007; Amato et al., 2018) and corresponded to an equivalent dose for humans of 80 mg/Kg (Reagan-Shaw et al., 2008).

The aqueous LG solution administered by gavage had a volume of 1 ml and a concentration of 83 mg LG/ml. Assuming an average mouse body weight of 20 g, this dose corresponded to 4.15 g LG/Kg of body weight (human equivalent dose, 340 mg LG/Kg of body weight).

### IOP Measurements and Pattern-electroretinography (PERG)

At baseline and after 1 and 2 mo from the beginning of treatment with LG, mice were dark adapted overnight and anesthetized by intraperitoneal injection of avertin (1.2% tribromoethanol and 2.4% amylene hydrate in distilled water, 0.02 ml/g body weight: Sigma-Aldrich). Anesthetized mice underwent IOP measurement after being positioned on a soft pad. The induction–impact tonometer (Tonolab Colonial Medical Supply, Franconia, NH, United States) was fixed by clamps on an adjustable stand and oriented with the probe aligned with the eye optical axis at a 1–2-mm distance. Five consecutive recordings were averaged to obtain a reliable measure of the IOP. At the end of the IOP assessment, eyes were instilled with saline solution to avoid corneal dryness. Then, the mice were transferred on a custom-made restrainer with unobstructed vision for the assessment of PERG. PERG was recorded from the right eye by means of a silver-silver chloride recording electrode configured to a semicircular loop of about 2 mm radius positioned on the corneal surface. Two stainless-steel needles positioned in the mouse cheek and at the tail root were used as reference and ground electrodes, respectively. The visual stimulus consisted in 98% contrast-reversing bars with 0.05 cycles/deg spatial frequency and 1 Hz temporal frequency delivered at 20 cm distance through a light emitting diode display with a mean luminance of 50 cd/m^2^. PERG signals were acquired using a commercially available PERG system (SB700 Advanced; Nikon-Europe, Amsterdam, Netherlands), amplified (10,000-fold) and band-pass filtered (1–30 Hz). Signals deriving from two consecutive recording protocols (PERG-rec1 and PERG-rec2; 300 pattern reversal stimulations each) were superimposed to confirm consistency and averaged to minimize noise. Individual averaged responses (hereinafter referred to as “PERG”) were analyzed by retrieving the signal amplitude and the implicit time after detecting the positive peak and the negative trough of the waveform (typically the P1 peak to the N2 trough). Since the recognition of the early negative component of the PERG waveform (N1) is classically ambiguous in D2 mice, we preferred to consider the P1-N2 amplitude as a reliable measure of PERG amplitude together with the time-to-P1 peak as PERG latency, as previously reported (Saleh et al., 2007). The test-retest reliability, calculated as the individual mean difference between PERG amplitudes of two consecutive recordings (|PERG-rec1 amplitude–PERG-rec2 amplitude|), was performed at the baseline and at the endpoint to estimate the interindividual variability.

### Immunofluorescence

Retinas were isolated and immersion fixed for 2 h at 4°C in 4% paraformaldehyde in 0.1 M phosphate buffered saline (PBS), and then stored at 4°C in 25% sucrose in 0.1 M PBS. The immunostaining of RGCs and astrocytes was performed by incubating the retinas with the guinea pig polyclonal antibody directed to RNA-binding protein with multiple splicing (RBPMS, ABN1376, dilution 1:100; Merck, Darmstadt, Germany), a ganglion cell marker (Rodriguez et al., 2014), and the rabbit monoclonal antibody directed to GFAP (ab207165, dilution 1:400; Abcam, Cambridge, United Kingdom) in PBS containing 5% bovine serum albumin (BSA) and 2% TritonX-100. After overnight incubation, the retinas were rinsed in PBS and incubated with FITC-conjugated anti-guinea pig secondary antibody (F6261; Merck) or AlexaFluor 555-conjugated anti-rabbit secondary antibody (A-32727; Molecular Probes, Eugene, OR, United States) diluted 1:200 in PBS containing 5% BSA and 2% TritonX-100. Finally, the retinas were rinsed in PBS and flat mounted on polarized glass slides with the RGC layer facing up. Images were acquired using an epifluorescence microscope (Ni-E; Nikon-Europe) equipped with a digital camera (DS-Fi1c camera; Nikon-Europe). Image sampling was performed in order to obtain four radial tiles (440 × 330 µm) in central and peripheral retina (center: 500 µm from the optic nerve head; periphery: 500 µm far from the peripheral edge). RGC density was measured in D2 retinas at baseline (D2b), in D2 retinas at the endpoint (D2e), and in D2+LG retinas as the average of the number of RBPMS immunopositive somata per mm^2^. The GFAP immunostaining was quantified as the average of the mean gray levels recorded in the sampled areas, after normalization to background, using ImageJ software (U. S. National Institutes of Health, Bethesda, Maryland, United States). The involvement of Müller glia activation was highlighted in virtual z-stack projections (150 µm) following image convolution filtering and 3D reconstruction with ImageJ. Finally, the concurrence between RGC loss and glial activation was analyzed by correlating the values of RBPMS and GFAP immunostainings.

### Quantitative Real-Time PCR

Quantitative real-time PCR (qPCR) was used to determine the expression of oxidative stress markers, including nuclear factor erythroid 2–related factor 2 (Nrf2), heme oxygenase-1 (HO-1), superoxide dismutase 2 (SOD-2), and glutamate-cysteine ligase catalytic subunit (GCLC), or of inflammatory markers, including interleukin 1 beta (IL-1β), interleukin 6 (IL-6), tumor necrosis factor alpha (TNF-α), and ionized calcium binding adaptor molecule 1(Iba1). Ribosomal protein L13A mRNA (Rpl13a) was used as an endogenous control. In all the experiments, three independent samples from each experimental group were analyzed. Total RNA was extracted and purified using RNeasy Mini Kit (Qiagen, Hilden, Germany). Then it was resuspended in RNAse-free water, and quantified by spectrophotometry (BioSpectrometer basic; Eppendorf AG, Hamburg, Germany). Starting from 1 µg of total RNA, first-strand cDNA was generated using a QuantiTect Reverse Transcription Kit (Qiagen). qPCR was performed using SsoAdvanced Universal SYBR Green Supermix on a CFX Connect Real-Time PCR Detection System provided with the software CFX manager (Bio-Rad Laboratories, Hercules, CA, United States). Primer sets were designed to hybridize to unique regions of the appropriate gene sequence according to published mouse cDNA sequences in the GenBank database (Table 1). Expression levels were quantified with the ∆∆Ct method.

**TABLE 1:** Primer sequences.

Gene	Forward primer (5′-3′)	Reverse Primer (5′-3′)
Nrf2	TCT​TGG​AGT​AAG​TCG​AGA​AGT​GT	GTT​GAA​ACT​GAG​CGA​AAA​AGG​C
HO-1	AAG​CCG​AGA​ATG​CTG​AGT​TCA	GCG​TGT​AGA​TAT​GGT​ACA​AGG​A
SOD-2	CAG​ACC​TGC​CTT​ACG​ACT​ATG​G	CTC​GGT​GGC​GTT​GAG​ATT​GTT
GCLC	GGG​GTG​ACG​AGG​TGG​AGT​A	GTT​GGG​GTT​TGT​CCT​CTC​CC
IL-1β	CCA​AGC​CTT​ATC​GGA​AAT​GA	TTG​TCG​TTG​CTT​GGT​TCT​CC
IL-6	GCC​TTC​CCT​ACT​TCA​CAA​GTC	AGT​GCA​TCA​TCG​TTG​TTC​ATA​C
TNF-α	GCC​TCT​TCT​CAT​TCC​TGC​TTG	CAC​TTG​GTG​GTT​TGC​TAC​GAC
Iba1	CGA​ATG​CTG​GAG​AAA​CTT​GG	AGCCCCACCGTGTGACAT
Rpl13a	CAC​TCT​GGA​GGA​GAA​ACG​GAA​GG	GCA​GGC​ATG​AGG​CAA​ACA​GTC

### Ultra-high Performance Liquid Chromatography and Mass Spectrometry (UHPLC-MS)

The main components of LG were determined in the LG powder (1 mg sample) using the same procedure as described below for the retinas. The most abundant were gallic acid, 4-hydroxybenzoic acid, quercetin, and nicotinamide (Table 2), therefore these metabolites were chosen as the LG components to be determined in the retina. To this aim, LG was administered to the mice by gavage and the retinas were dissected at different time points. The two retinas from each mouse were pooled together and they were resuspended in 0.2 ml of ice cold ultra-pure water (18 MΏ-cm) to lyse the cells. Three independent samples were analyzed for each time point. The tubes were plunged into dry ice or in a circulating bath at −25°C for 0.5 min and then into a water bath at 37°C for 0.5 min. Subsequently, 0.6 ml of −20°C methanol and then 0.4 ml of −20°C chloroform were added to each tube. The tubes were mixed every 5 min for 30 min, then they were centrifuged at 1000 × g for 1 min at 4°C, before being transferred to −20°C for 2–8 h. After centrifugation at 15,000 × g for 10 min at 4°C, the supernatants were collected and dried. Finally, the dried samples were resuspended in 0.1 ml of water and transferred to glass autosampler vials for LC-MS analysis. The supernatants were injected (20 μl) into a UHPLC system (Ultimate 3000; ThermoFisher Scientific, Waltham, MA, United States) and run in negative ion mode for determination of 4-hydroxy benzoic acid and in positive ion mode for the other. A ReproSil C18 column (2.0 mm × 150 mm, 2.5 μm; Dr Maisch, Ammerbuch-Entringen, Germany) was used for metabolite separation. Chromatographic separations were achieved at a column temperature of 30°C and flow rate of 0.2 ml/min. For positive ion mode (+) MS analyses, a 0–100% linear gradient of solvent A (ddH_2_O, 0.1% formic acid) to B (acetonitrile, 0.1% formic acid) was employed over 20 min returning to 100% A in 3 min. For negative ion mode chromatographic separations A 0–100% linear gradient of solvent A (double-distilled 18 MΏ-cm water, 10 mm ammonium acetate) to B (100% acetonitrile, 10 mm ammonium acetate) was employed over 20  min, returning to 100% A in 3 min and a -min post-time solvent A hold. The UHPLC system was coupled online with a mass spectrometer Q Exactive (ThermoFisher) scanning in full MS mode (2 μ scans) at 70,000 resolution in the 60–1000 m/z range. Data files were processed by MAVEN. 8.1 (http://genomics-pubs.princeton.edu/mzroll/) upon conversion of raw files into mzXML format through MassMatrix (Yellow Springs, OH, United States). Standard curves were obtained with several calibration points (2–0.00002 mg) of gallic acid, 4-hydroxybenzoic acid, quercetin, and nicotinamide analytical standards (Sigma Aldrich, St Louis, MO, United States).

**TABLE 2:** Amounts of the selected compounds in LG and in the administered dose of LG.

Compound	mg/mg LG	mg/administered dose of LG
Gallic acid	0.1928 ± 0.0418	16.0100 ± 3.4700
Hydroxy benzoic acid	0.0163 ± 0.0003	1.3530 ± 0.0289
Quercetin	0.0023 ± 0.0004	0.1897 ± 0.0364
Nicotinamide	0.0018 ± 0.0005	0.1501 ± 0.0432

### Statistical Analysis

For the analysis of LG bioactivity, the sample size (*n* = 6) was calculated in order to ensure an effect size on the main output parameter (PERG amplitude) as narrow as 1.5 µV with an α error of 0.05 and 1-β power of 0.08. Data displaying the effect of one categorical variable (treatment) were analyzed using unpaired *t*-test. Data describing the effect of two combined categorical variables (treatment and time) were analyzed with two-way ANOVA with Bonferroni *post-hoc* test for multiple comparisons. The correlation between two dependent variables was tested with the two-tailed Pearson’s test for linear regression analysis and performing ANCOVA for the comparison between groups identified according to a categorical variable. Data were expressed as mean ± SEM of the respective *n* values (Prism 8; GraphPad software, San Diego, CA, United States). Differences with *p* < 0.05 were considered significant.

## Results

### LG Does Not Affect IOP

**FIGURE 1:**
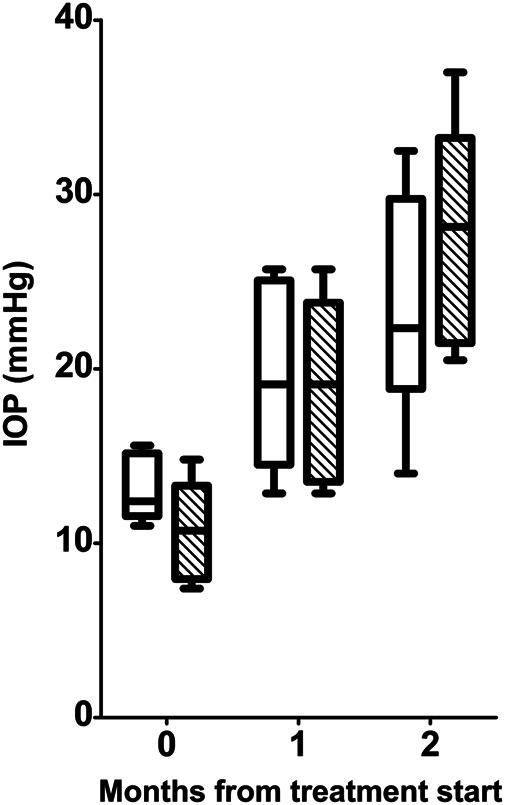
Longitudinal analysis of IOP levels in D2 (white boxes) and D2+LG (dashed boxes). Box plots describe the statistical distribution of data deriving from the average of IOPs measured in individual right and left eyes of *n* = 6 mice. Statistical differences were tested using two-way ANOVA with Bonferroni *post-hoc* test. The effect of time was highly significant (*p* < 0.0001), while the effect of LG at each time was not significant (*p* = 0.6782).

As shown in Figure 1, during the 2-mo period of the experiment, the IOP of D2 mice increased from 13.13 ± 1.85 mmHg to 23.50 ± 6.38 mmHg, while the IOP of D2+LG mice went from 10.78 ± 3.08 mmHg to 27.93 ± 6.50 mmHg (effect of time, *p* < 0.0001). No differences were observed in IOP levels at any time between D2 and D2+LG mice (effect of LG, *p* = 0.6782).

### LG Improves the PERG Responses

**FIGURE 2:**
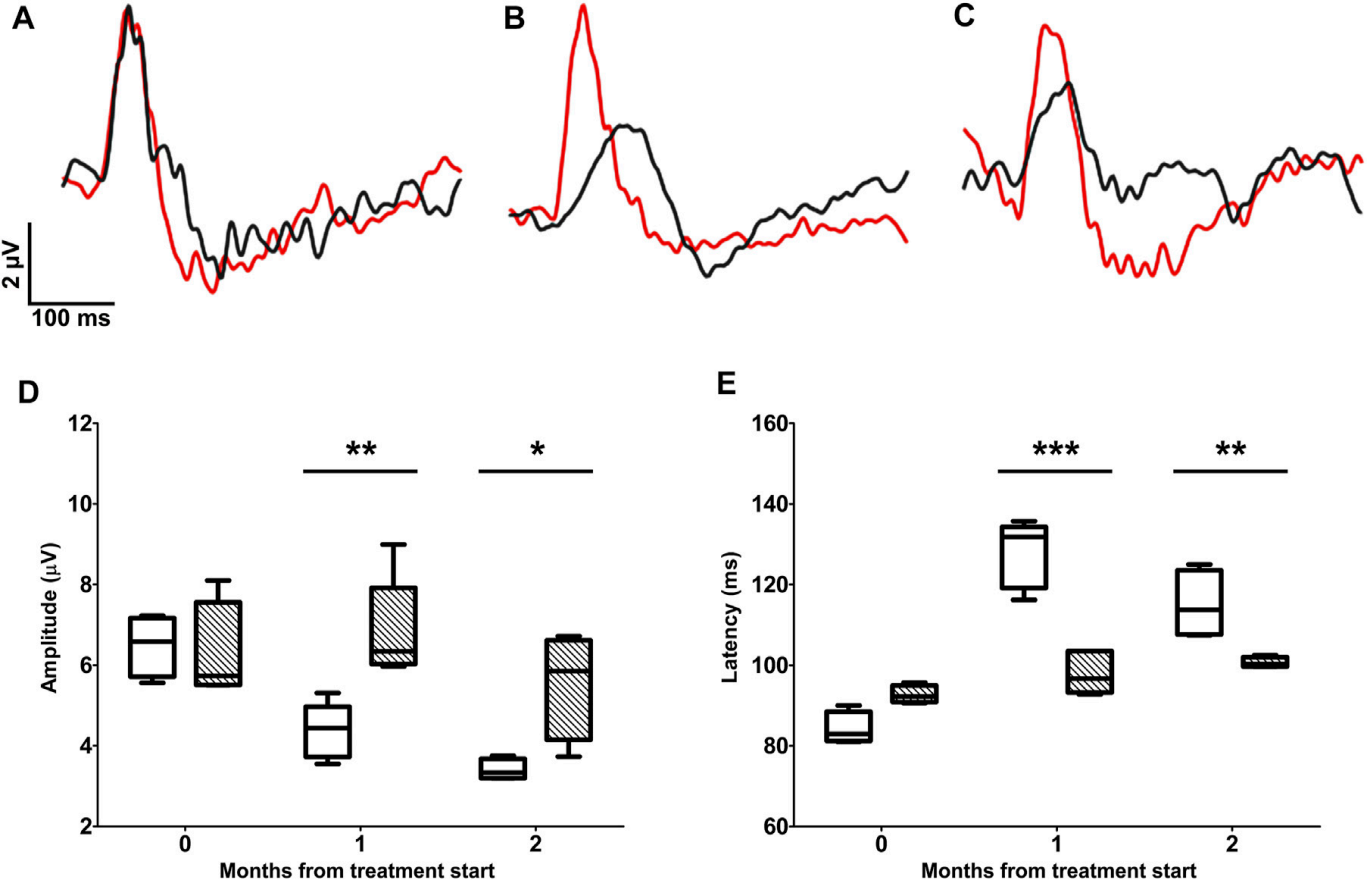
Longitudinal evaluation of RGC activity using PERG. PERG responses were recorded before the beginning of the treatment **(A)** and after 1 **(B)** or 2 **(C)** months of treatment in D2 (black traces) and D2+LG (red traces) mice. PERG responses were analyzed by retrieving the peak-to-trough amplitude and time-to-peak latency, as shown in the inset in **(A)**. The distribution of PERG amplitudes **(D)** and PERG latencies **(E)** in D2 (white boxes) and D2+LG (dashed boxes) deriving from *n* = 6 mice are shown by box plots. Statistical differences were tested using two-way ANOVA with Bonferroni *post-hoc* test. *p < 0.05, **p < 0.01, ***p < 0.001.

The test-retest analysis between individual PERG-rec1 and PERG-rec2 displayed an amplitude interindividual variability of 0.58 ± 0.05 µV (95% CI 0.42–0.73) in D2 and 0.59 ± 0.03 µV (95% CI 0.49–0.70) in D2 + LG retinas at the baseline. Similarly, at the endpoint the variability was 0.56 ± 0.06 µV (95% CI 0.34–0.77) in D2 and 0.57 ± 0.09 µV (95% CI 0.31–0.83) in D2+LG retinas. By comparing the interindividual variability retrieved in both groups between baseline and endpoints, we did not observe any significant effect of either the time (*p* = 0.77) or the treatment (*p* = 0.85). Therefore, since the interindividual variability was constant among experimental groups over time, we considered the averages between PERG-rec1 and PERG-rec2 waveforms in order to minimize the noise contribution in the analysis of RGC activity (Figures 2A–C). PERG responses were similar in D2 and D2+LG mice at the baseline (PERG amplitude, *p* > 0.99; PERG latency, *p* = 0.1533). During the follow-up period, D2 mice displayed a time-dependent decline in PERG amplitude resulting in a ~ 53% loss at the endpoint compared to baseline. The decrease in PERG amplitude appeared less evident in the D2+LG group than in the D2 group (effect of LG, *p* = 0.013), since the values remained around baseline levels after 1 mo of treatment and were only partially reduced after 2 mo (Figure 2D). Thus, LG treatment resulted in a significant maintenance of the endpoint PERG amplitude compared to the D2 group (D2 amplitude: 3.4 ± 0.38 µV; D2+LG amplitude: 5.5 ± 0.66 µV; *p* = 0.019). PERG latency (Figure 2E) was increased over the period under investigation in both groups (effect of time, *p* < 0.001). However, PERG latency recorded in D2+LG mice after 1 and 2 mo was lower than in the D2 group (effect of LG, *p* < 0.001), thus resulting partially preserved at the endpoint (D2 latency: 114.99 ± 4.26 ms; D2+LG latency: 100.68 ± 0.64 ms; *p* = 0.0067).

### LG Reduces Oxidative Stress and Inflammatory Markers

**FIGURE 3:**
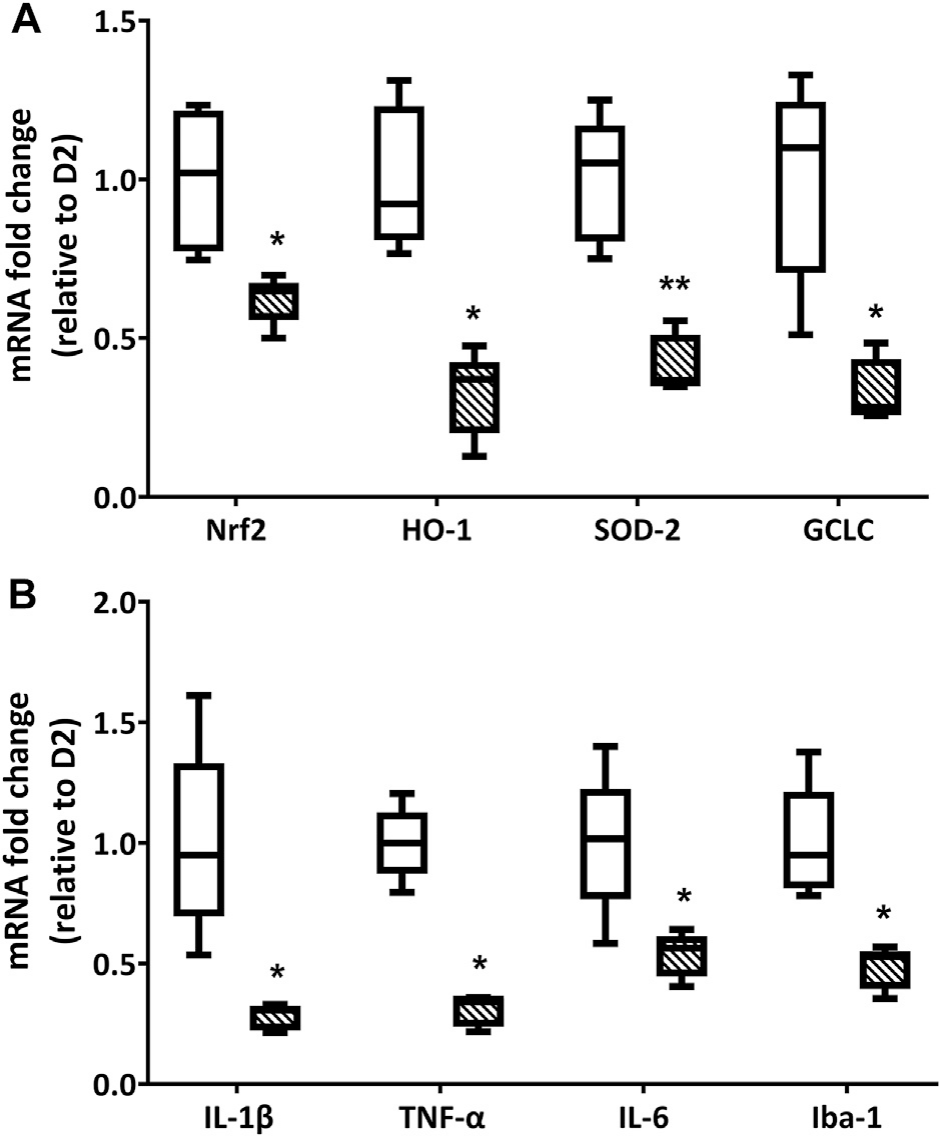
Endpoint qPCR analysis for the expression of oxidative stress **(A)** and inflammation-related **(B)** markers in D2 (white box) and D2+LG (dashed boxes) groups after 2 mo of treatment. Box plots describe the statistical distribution of *n* = 6 independent retinas. Differences between groups were tested using two-tailed *t*-test. *p < 0.05, **p < 0.01 vs D2.

The treatment with LG produced a significant decrease in the expression of oxidative stress-related genes. Indeed, as shown in Figure 3A, the qPCR analysis revealed an expression of Nrf2 mRNA that was slightly above 50% of that measured in D2 retinas, while the expression levels of HO-1, SOD-2, and GCLC mRNAs were all less than 50% of those detected in D2 retinas. Similarly, the mRNAs of inflammatory markers (Figure 3B) were also reduced to about 50% (IL-6 and Iba-1) or less (IL-1β and TNF-α) of the values measured in the retinas of D2 mice.

### LG Preserves RGC Density

**FIGURE 4:**
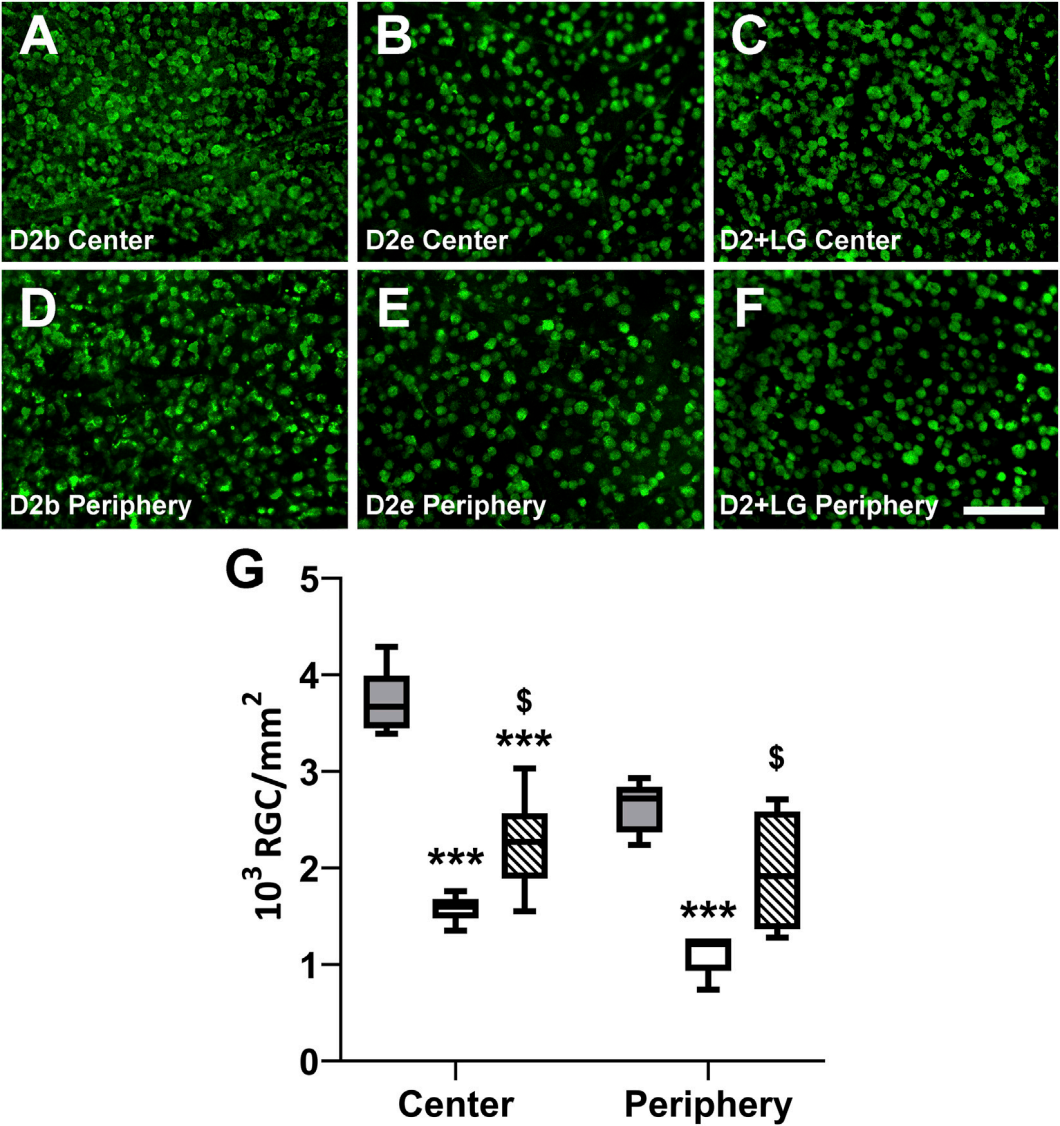
Baseline and endpoint densitometric analysis of RBPMS-immunostained RGCs in whole mount retinas. Immunofluorescence photomicrographs show representative RBPMS immunostaining in central and peripheral areas of D2b, **(A,D)**, D2e **(B,E)** and D2+LG **(C,F)** retinas. Scale bar, 100 μm. **(G)** analysis of RBPMS positive cell density in D2b (gray boxes), D2e (white boxes), and D2+LG (dashed boxes) retinas. Box plots describe the statistical distribution of n = 4 independent retinas. Data were analyzed using two-way ANOVA with Bonferroni *post-hoc* test. *p < 0.05 vs. D2.

The RGC density was evaluated as the number of RBPMS immunopositive cells/mm^2^ sampled in peripheral and central areas of D2b, D2e, and D2+LG retinas (Figures 4A–F). The RBPMS immunopositive cell density was influenced by the sampling location, resulting lower in the retinal periphery than in the retinal center (*p* = 0.0397). As shown in Figure 4G, in D2 retinas there was a dramatic decrease of immunolabeled RGC density with a loss of about 57% from baseline to endpoint both in central and in peripheral retinal locations. In D2+LG retinas, the decrease of RGC density reached 39% in central retina and 26% in the periphery. The retinas belonging to the D2+LG group displayed a significantly higher RBPMS immunopositive cell density than those of the D2e group (center, *p* = 0.0106; periphery, *p* = 0.0114). Interestingly, there was no statistically significant difference between peripheral RGC density in D2b and in D2+LG retinas.

### LG Attenuates Macroglial Activation

**FIGURE 5:**
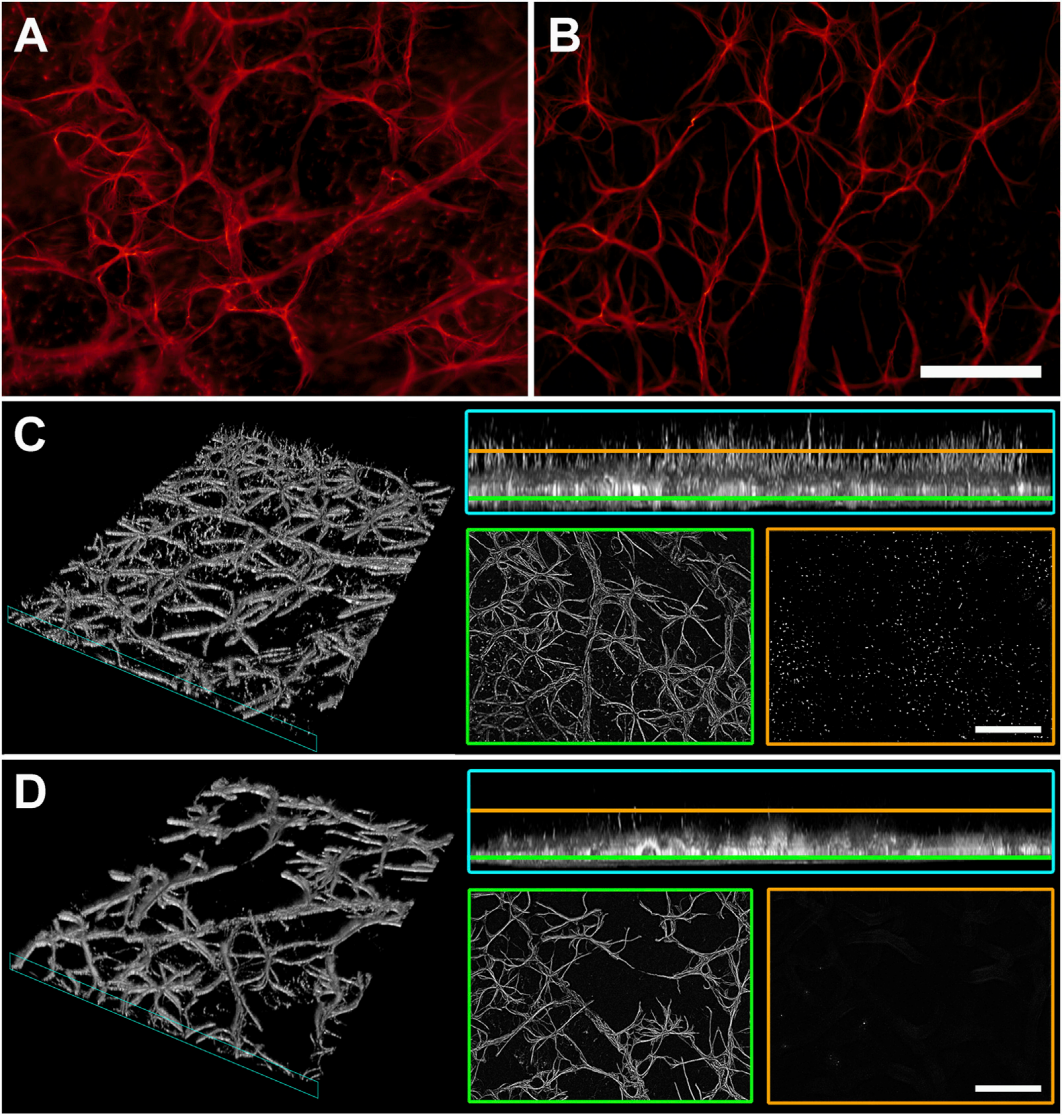
GFAP immunostaining in whole mount retinas of D2 **(A)** and D2+LG **(B)** mice for the evaluation of macroglia activation. **(C**,**D)** virtual 3D reconstructions of 70 µm z-stacks of D2 and D2+LG retinas, respectively, displaying GFAP-immunopositive processes in the retinal thickness (cyan insets) and surface projections distancing 10 µm (yellow) and 45 µm (green) from the uppermost focal plan. Scale bars, 100 µm.

**FIGURE 6:**
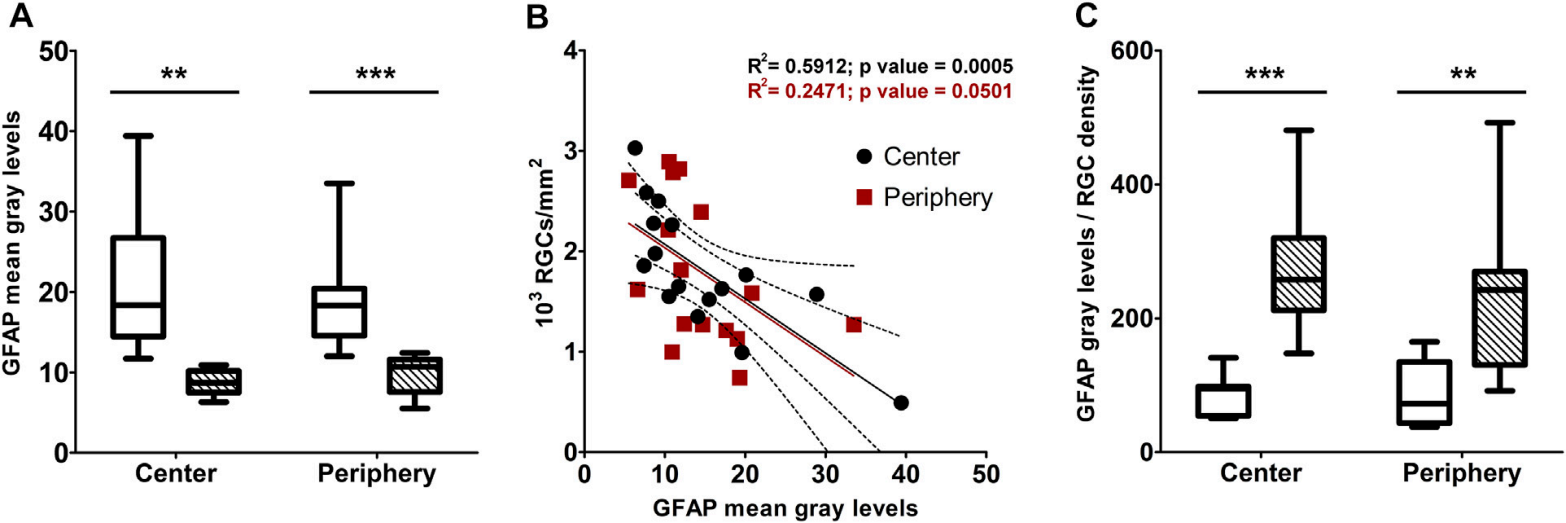
**(A)** densitometric analysis of GFAP immunostaining in central and peripheral areas of D2 (white box) and D2+LG (dashed boxes). **(B)** correlation of GFAP immunofluorescence levels with the correspondent RGC density per optical filed in central and peripheral retinal areas. **(C)** ratio between GFAP immunostaining and RGC density in central and peripheral retinal areas of D2 (white box) and D2+LG (dashed boxes) mice. Box plots describe the statistical distribution of *n* = 4 independent retinas. Data were analyzed using two-way ANOVA with Bonferroni *post-hoc* test. **p < 0.01, ***p < 0.001.

Macroglial reactivity was analyzed using GFAP immunolabeling of retinas of the D2 and D2+LG groups. GFAP immunopositive astrocytes in D2 retinas displayed a typical reactive phenotype with slightly hypertrophic and poorly organized branching. In addition, a prominent presence of GFAP immunopositive profiles with a punctate appearance was evident among astrocytic processes (Figure 5A). In contrast, astrocytes in D2+LG retinas displayed thinner processes and a more organized arborization, with more sporadic and less evident GFAP immunopositive profiles among astrocytic processes (Figure 5B). Interestingly, the z-stack projection of the analyzed areas in the D2 retinas revealed that the GFAP immunopositive puncta corresponded to vertically oriented processes spanning the thickness of the retina and reminding the typical pattern of activated Müller cells (Figure 5C). As expected, these processes were less evident in D2+LG retinas (Figure 5D). Accordingly, the levels of GFAP immunostaining in the D2+LG retinas were significantly lower than those in D2 retinas both in central and in peripheral retinal regions (Figure 6A). In addition, the RBPMS immunopositive cell densities negatively correlated with the GFAP immunofluorescence levels (center, *p* = 0.0005; periphery, *p* = 0.05; Figure 6B) and the ratio between the two parameters was increased in D2+LG retinas as compared to D2 retinas (*p* < 0.0001; Figure 6C).

### Characterization of LG Components and Their Detection in the Retina

**FIGURE 7:**
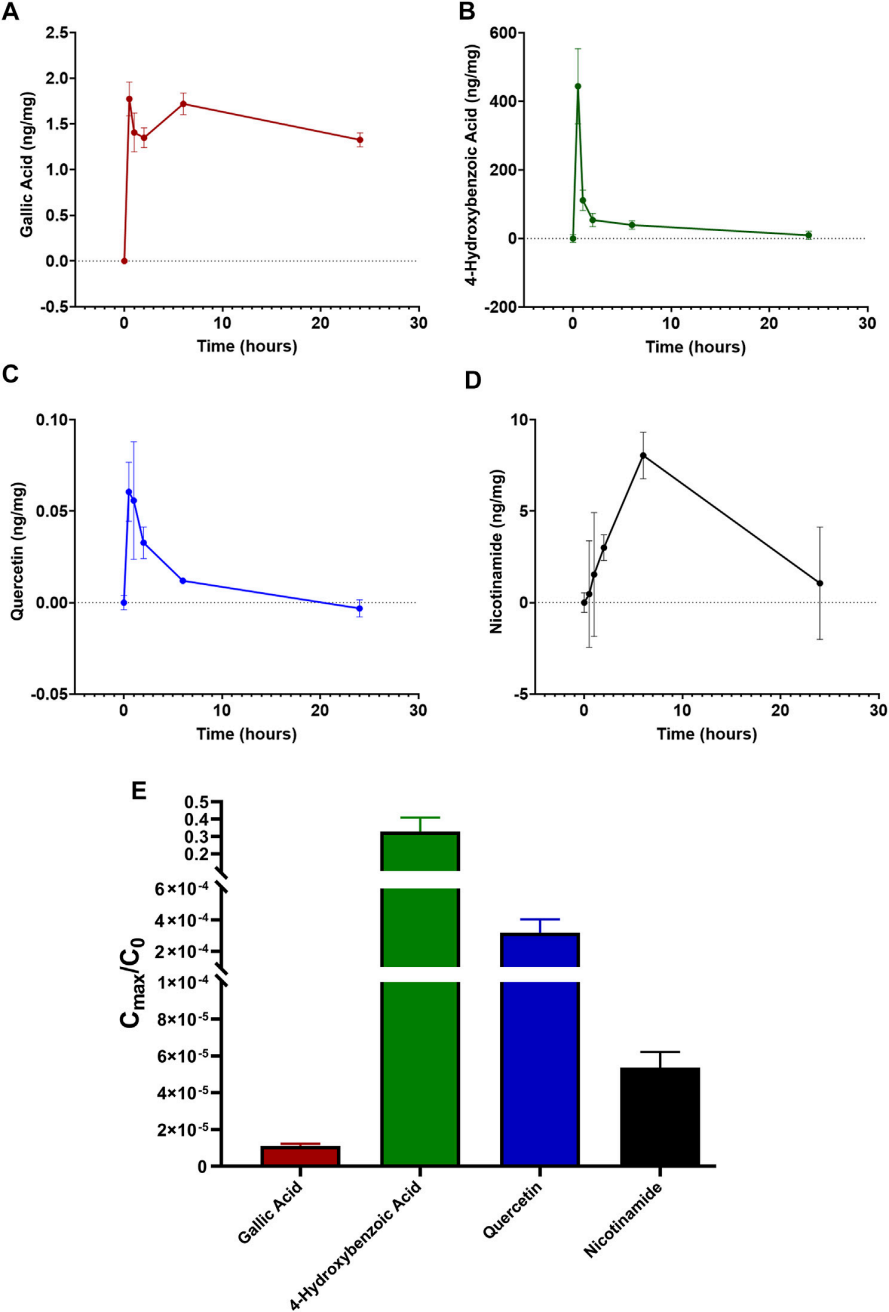
Time-dependent retinal profile of gallic acid **(A)**, 4-hydroxybenzoic acid **(B)**, quercetin **(C)**, and nicotinamide **(D)** amount after administration of 1 mg LG by oral gavage. **(E)** corresponding ratios calculated as the maximal amount of each compound reaching the retina (C_max_) divided by the initial amount of the same compound administered in 1 mg of LG (C_0_). Data are expressed as mean ± SEM of *n* = 3 independent samples.

The characterization of the main LG components has been provided in previous papers (Longo et al., 2007; La Marca et al., 2013; Lucchesi et al., 2014; Gabriele et al., 2018). Our findings were in line with these data and indicated a series of metabolites as the main bioactive components of the LG solution used in the present studies. We choose four of them, namely gallic acid, 4-hydroxybenzoic acid, quercetin, and nicotinamide, for tracing their presence in the retina after oral LG administration. Gallic acid was the compound with the highest amount within the LG solution and, consequently, within the dose of LG administered to the mice by gavage (Table 2). As shown in Figures 7A–D, all four compounds were detected in the retina after LG administration and the variations of their concentrations in the retinal tissue were characterized by specific time courses. In particular, the maximum concentration of nicotinamide was observed after 6 h, while those of the other compounds were reached as soon as 30 min after LG administration (the actual values are reported in Table 3). In addition, levels that were still in the range of its maximum concentration were observed for gallic acid after 24 h from LG administration (the longest time period investigated). At this time, detectable, although low, levels of nicotinamide were also measured. In contrast, quercetin levels decreased quickly within 2 h and even quicker was the decrease of the levels of 4-hydroxybenzoic acid after a large peak recorded at 30 min. Considering the ratio between the amount in the administered LG and the maximum amount recovered in the retina (Figure 7E), it turned out that a very high proportion (around 38%) of the 4-hydroxybenzoic acid administered with LG could reach the retina, while only 0.01% of the ingested gallic acid could be recovered. The other values were, on average, 5.55% for quercetin and 8.76% for nicotinamide.

**TABLE 3:** Pharmacokinetic parameters of the selected compounds in the retina.

Compound	*C*_*max*_ (ng/mg*)	*T*_*max*_ *(h)*	*AUC*_*0-24h*_ (ng·h/mg*)
Gallic acid	1.770 ± 0.184	0.5	36.150 ± 2.284
Hydroxy benzoic acid	444.250 ± 109.713	0.5	958.500 ± 291.500
Quercetin	0.061 ± 0.016	0.5	0.263 ± 0.049
Nicotinamide	8.040 ± 1.274	6	106.900 ± 52.210

C_max_, maximum concentration; T_max_, time of C_max_; AUC_0–24h_, area under the concentration versus time curve from 0 to 24 h; *mg of retinal tissue (wet weight).

## Discussion

The present research strongly suggests that the administration of a dietary supplement may reduce significantly the pathologic changes associated to glaucoma and ameliorate visual function. Many substances of natural origin have been tested for their protective roles against glaucoma progression, although studies at the clinical level have not confirmed these potential benefits for the human pathology yet (Hernández-Rabaza et al., 2019; Adornetto et al., 2020). Nevertheless, nutritional supplements with antioxidant, anti-inflammatory, and/or neuroprotective features still promise important advancements when their properties are better investigated at the clinical level and their bioavailability is improved. In particular, the maintenance of RGC function, as evaluated with PERG analysis, together with the prevention of RGC loss, indicate a dual effect of neuroenhancement and neuroprotection exerted by LG in glaucoma.

### The Potential of LG for the Treatment of Retinal Diseases

In the present work, a specific nutraceutical, LG, was investigated. From the results, we can infer that it provides significant neuroenhancement and neuroprotection against the structural and functional retinal damage induced by glaucoma and that at least some of its components reach the retina after oral administration. We have recently tested the effectiveness of LG in protecting the retina in an experimental model of diabetic retinopathy and we found that it could inhibit oxidative stress, apoptosis, and vascular endothelial growth factor expression. At the same time, it also prevented blood-retinal barrier damage, reduced the levels of inflammatory markers and partially restored visual function (Amato et al., 2018). This wide range of positive effects could be explained with the powerful antioxidant effects of LG, assuming that most, if not all, pathologic changes in the diabetic retina are induced by an initial phase of oxidative stress [*see* (Rossino and Casini, 2019; Rossino et al., 2019) for discussion]. A similar interpretation may be proposed to explain the protective effects of LG in glaucoma. Indeed, although IOP elevation is known as one of the most recurrent risk factors for glaucoma (Nickells et al., 2012) and is the main target of pharmaceutical treatments (Stein et al., 2021), often it could manifest only at late stages of the disease or may not occur, as in the case of normotensive glaucoma (Weinreb et al., 2014). Similar to diabetic retinopathy, oxidative stress is likely to represent a major trigger also for glaucoma (Domènech and Marfany, 2020; Garcia-Medina et al., 2020; Harada et al., 2020; Wang et al., 2020), and LG, which does not have any effect on the rise of IOP characteristic of D2 mice, is likely to exert its protective effect thanks to its powerful antioxidant properties.

The antioxidant effects of LG may be due to direct radical scavenging and/or strengthening of antioxidant defenses through the activation of Nrf2 and the expression of antioxidant enzymes (La Marca et al., 2013). Similar to our previous findings in a diabetic retinopathy model (Amato et al., 2018), LG treatment did not result in an increase of HO-1, SOD-2 or GCLC expression, indicating that the antioxidant effect of LG is likely to be due to its radical scavenging properties. This is in line with the observed decrease of Nrf2 mRNA: we may interpret these data assuming that radical scavenging by LG reduces the level of oxidative stress, which, in turn, determines a decrease of Nrf2 expression (and likely a decreased Nrf2 nuclear translocation, as observed in diabetic rat retinas (Amato et al., 2018)), which results in decrease of antioxidant enzyme expression.

As reported in primary rat hepatocytes (La Marca et al., 2013), in human endothelial progenitor cells (Giusti et al., 2017), and in diabetic rat retinas (Amato et al., 2018), a further effect of LG is inhibition of the nuclear translocation of nuclear factor kappa-light-chain-enhancer of activated B cells (NF-kB), an oxidant-sensitive transcription factor that regulates the expression of factors involved in inflammation. Thanks to this interaction with NF-kB and in accordance with the reported relationships between oxidative stress and inflammation (Gill et al., 2010; Lugrin et al., 2014), the reduced oxidative stress is likely to be responsible for the observed decrease of the expression of inflammatory markers and of macroglial activation (Subirada et al., 2018; Domènech and Marfany, 2020). In addition, the finding of a negative correlation, both in central and in peripheral retinal regions, between RGC density and macroglial activation (evaluated with mean GFAP immunofluorescence levels) supports a tight relationship between intensity of the inflammatory response and the rate of RGC loss. Therefore, the more favorable conditions in D2+LG retinas compared to those in D2 retinas, with lower levels of both oxidative stress and inflammation, would support RGC survival and maintenance of retinal function. In particular, our results indicate that a large proportion of RGCs is lost during the 2 mo of the experiments, probably due to the dramatic increase in IOP levels in the same period. The treatment with LG saves a significant portion of these RGCs, and this is particularly relevant in peripheral retina, where LG treatment results in virtually complete rescue of the RGCs. According to these observations, both preclinical and clinical studies have reported RGC loss and PERG defects in glaucomatous retinas, which were protected by antioxidant treatments obtained with nutritional supplements (Parisi et al., 2014; Cammalleri et al., 2020; Himori et al., 2021; Oddone et al., 2021).

Together, these observations strongly indicate LG as a basis for the potential treatment of a variety of retinal pathologies, also considering that the use of LG on human subjects is facilitated by the fact that it has been recognized and registered by the Italian Ministry of Health as a nutritional supplement.

### LG Components Reaching the Retina After Oral Administration

The characterization of LG components has been provided in different papers (Longo et al., 2007; La Marca et al., 2013; Lucchesi et al., 2014; Gabriele et al., 2018). In general, a fermentation process induces the production or extraction of bioactive compounds from several natural sources. In particular, in the preparation of LG it determines an increase in both polyphenol content and antioxidant capacity. Indeed, the fermentative process not only decreases anti-nutrients, improves protein digestibility, and reduces allergenicity, but also provides a higher content of bioactive compounds and antioxidant activity (Gabriele and Pucci, 2017). The compounds whose levels are mostly increased in LG after fermentation are gallic acid, linolenic acid, linoleic acid, and lipoic acid (La Marca et al., 2013; Gabriele et al., 2018). Of them, gallic acid is likely to be the most abundant metabolite in the hydrophilic fraction that we used in our experiments, while other highly present components were 4-hydroxybenzoic acid, quercetin, and nicotinamide.

Bioavailability is a pharmacokinetic term referring to the fraction of bioactive compound that reaches the blood circulation without undergoing alterations. In general, ingested natural substances of vegetal origin are subjected to profound alteration of their structure due to digestive processes in the gastrointestinal tract and hepatic metabolism and only a small fraction of them can finally reach the various tissues of the body, therefore investigations to improve this aspect are of fundamental importance [*see* (Rossino and Casini, 2019) for review]. We have evaluated the fraction of LG metabolites that reach the retina since this is the most valuable information for further studies on LG metabolites to increase their retinal content and improve their efficacy. Interestingly, our data show very different behaviors for some of the metabolites that were analyzed: although the retinal concentration of gallic acid remained stable for 24 h post administration, the fraction of this metabolite reaching the retina was minimal, while 4-hydroxybenzoic acid could reach the retina in relatively high amounts, but its levels were drastically decreased after only 1 h from ingestion. These differences are likely to depend on the size, chemical structure and lipophilicity of the compound (Del Amo et al., 2017) and constitute important factors to consider in designing drug delivery methods to improve retinal availability.

### Protective Actions of LG Components in Glaucoma

As noted above, LG is made of several different components, therefore the possibility exists that the effects of LG reported in this investigation may derive, at least in part, from components that are different from those analyzed here in detail, although they are likely to be present only in low amounts in the hydrophilic LG fraction used in our studies. For instance, fatty acids like linoleic and linolenic acids, as well as α-lipoic acid and vitamins have been identified in LG and they have been postulated to mediate antioxidant effects of LG in primary rat hepatocytes (La Marca et al., 2013). In any case, while considering this, we favor the idea that the effects of LG observed in the present study are due to the components that are present in highest amounts in the hydrophilic fraction, which include gallic acid, quercetin, 4-hydroxybenzoic acid, and nicotinamide.

Gallic acid is a phenolic acid known as a potent antioxidant molecule and a neuroprotectant (Daglia et al., 2014), while 4-hydroxybenzoic acid is one of the phenolic derivatives of benzoic acid, which are known for their antioxidant properties (Beata and Ivan, 2012). It is only slightly soluble in water, which may explain the relatively low amount of this compound, with respect to gallic acid, in the hydrophilic fraction of LG used in our studies. It has been shown that intraocular delivery in glaucomatous rabbit eyes of pilocarpine-loaded biodegradable thermogels functionalized with gallic acid to provide antioxidant capacity results in improved total ocular antioxidant status, enhancement of the retinal antioxidant defense system, and preservation of retinal histology and ERG responses (Lai and Luo, 2015; Chou et al., 2016; 2017). Similarly, a benzoic acid derivative used in a thermogel coloaded with pilocarpine has been reported to enhance antioxidant capacity and neuroprotection in rabbit eyes with glaucoma (Luo et al., 2020).

Quercetin is a flavonol found in vegetables and fruits, and its properties as a potential beneficial treatment for diabetic retinopathy as well as for other ocular diseases have been reviewed recently (Rossino and Casini, 2019; Zhao et al., 2021). Concerning glaucoma, quercetin has been found to reduce excitotoxic damage to RGCs (Zhou et al., 2019) and to improve mitochondrial function, RGC survival, and ERG responses (Gao et al., 2017) in rat models of ocular hypertension.

Nicotinamide is a form of vitamin B3 and a precursor of nicotinamide adenine dinucleotide (NAD^+^) and nicotinamide adenine dinucleotide phosphate, which are involved in different aspects of cellular metabolism (Amjad et al., 2021). Both clinical and experimental data suggest that NAD^+^ supplementation may be therapeutic in neurodegenerative retinal diseases such as age-related macular degeneration and glaucoma (Kouassi Nzoughet et al., 2019; Cimaglia et al., 2020; Hui et al., 2020). Supporting these observations, findings in D2 mice reported a significant decrease of retinal NAD^+^ levels with age, while oral administration of nicotinamide and/or gene therapy for the expression of a NAD^+^-producing enzyme reduced mitochondrial abnormalities and protected RGCs from degeneration (Williams et al., 2017; Williams et al., 2018). In addition, a recent work also showed that a nicotinamide-rich diet may efficiently rescue RGCs and mitochondria, and preserves flicker-induced PERG adaptation in D2 mice (Chou et al., 2020).

## Conclusion

The present study confirms and expands the notion that LG may reveal as a powerful therapeutic tool to treat glaucoma, diabetic retinopathy and, very likely, other sight-threatening retinal diseases. The data presented herein indicate that LG contains different metabolites with documented anti-glaucoma properties. Together with other metabolites, their co-occurrence in LG may provide an added value to their beneficial effects in glaucoma and other retinal diseases. A limitation of the present study could be the characterization of the LG components in the retina that needs to be further integrated with a full pharmacokinetic analysis. Concurrently, the challenge is to increase the amount of LG metabolites that may reach the retina after oral administration, a task that we plan to tackle in the near future using nanotechnology approaches.

## Data Availability

The raw data supporting the conclusions of this article will be made available by the authors, without undue reservation.
