# Correction: The potential of Lisosan G as a possible treatment for glaucoma

**DOI:** 10.3389/fphar.2026.1850441

**Published:** 2026-06-10

**Authors:** Rosario Amato, Maria Grazia Rossino, Maurizio Cammalleri, Anna Maria Timperio, Giuseppina Fanelli, Massimo Dal Monte, Laura Pucci, Giovanni Casini

**Affiliations:** 1 Department of Biology, University of Pisa, Pisa, Italy; 2 Interdepartmental Research Center Nutrafood “Nutraceuticals and Food for Health”, University of Pisa, Pisa, Italy; 3 Department of Ecological and Biological Sciences, University of Tuscia, Viterbo, Italy; 4 Department of Agriculture and Forest Sciences, University of Tuscia, Viterbo, Italy; 5 National Research Council, Institute of Agricultural Biology and Biotechnology (IBBA), Pisa, Italy

**Keywords:** inflammation, neuroprotection, nutraceuticals, oxidative stress, pattern electroretinogram

There was a mistake in Figure 4A as published. A preliminary version of the panel shown in Figure 4A was included erroneously in the final version of the figure. The corrected Figure 4 appears below.

**FIGURE 4:**
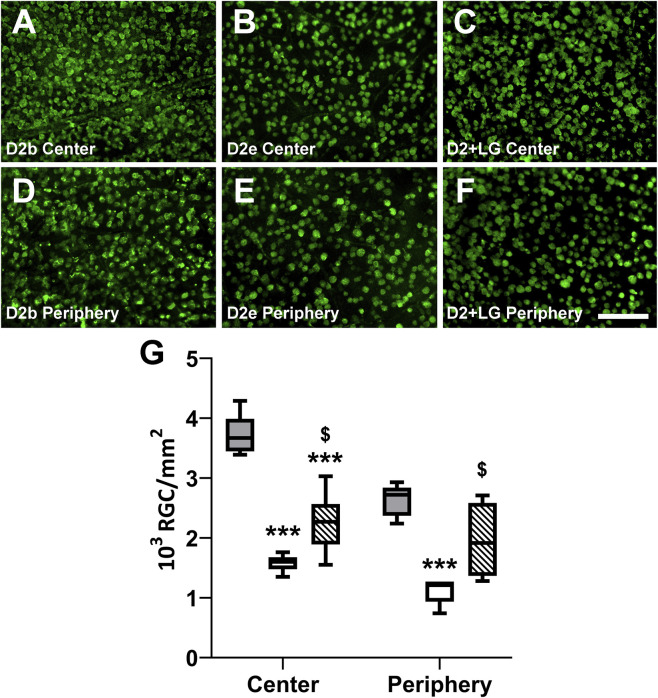
Baseline and endpoint densitometric analysis of RBPMS-immunostained RGCs in whole mount retinas. Immunofluorescence photomicrographs show representative RBPMS immunostaining in central and peripheral areas of D2b, **(A,D)**, D2e **(B,E)** and D2+LG **(C,F)** retinas. Scale bar, 100 μm. **(G)** analysis of RBPMS positive cell density in D2b (gray boxes), D2e (white boxes), and D2+LG (dashed boxes) retinas. Box plots describe the statistical distribution of n = 4 independent retinas. Data were analyzed using two-way ANOVA with Bonferroni *post-hoc* test. *p < 0.05 vs. D2.

The original article has been updated.

